# Identification of brain regions associated with working memory deficit in schizophrenia

**DOI:** 10.12688/f1000research.17731.1

**Published:** 2019-01-30

**Authors:** Indranath Chatterjee, Virendra Kumar, Sahil Sharma, Divyanshi Dhingra, Bharti Rana, Manoj Agarwal, Naveen Kumar

**Affiliations:** 1 Department of Computer Science, University of Delhi, Delhi, DELHI, 110007, India; 2 Department of NMR and MRI Facility, All India Institute of Medical Sciences, Delhi, DELHI, 110029, India; 3 Department of Computer Science, Hans Raj College, University of Delhi, Delhi, DELHI, 110007, India

**Keywords:** functional Magnetic Resonance Imaging, fMRI, Schizophrenia, Working Memory, Group Independent Component Analysis, Classification, Computer-aided Diagnosis

## Abstract

**Background:** Schizophrenia, a severe psychological disorder, shows symptoms such as hallucinations and delusions. In addition, patients with schizophrenia often exhibit a deficit in working memory which adversely impacts the attentiveness and the behavioral characteristics of a person. Although several clinical efforts have already been made to study working memory deficit in schizophrenia, in this paper, we investigate the applicability of a machine learning approach for identification of the brain regions that get affected by schizophrenia leading to the dysfunction of the working memory.

**Methods:** We propose a novel scheme for identification of the affected brain regions from functional magnetic resonance imaging data by deploying group independent component analysis in conjunction with feature extraction based on statistical measures, followed by sequential forward feature selection. The features that show highest accuracy during the classification between healthy and schizophrenia subjects are selected.

**Results:** This study reveals several brain regions like cerebellum, inferior temporal gyrus, superior temporal gyrus, superior frontal gyrus, insula, and amygdala that have been reported in the existing literature, thus validating the proposed approach. We are also able to identify some functional changes in the brain regions, such as Heschl gyrus and the vermian area, which have not been reported in the literature involving working memory studies amongst schizophrenia patients.

**Conclusions:** As our study confirms the results obtained in earlier studies, in addition to pointing out some brain regions not reported in earlier studies, the findings are likely to serve as a cue for clinical investigation, leading to better medical intervention.

## Introduction

Schizophrenia is a psychological disorder that involves auditory and visual hallucinations and delusions. A schizophrenia patient often shows symptoms such as disorganized thinking, difficulty in speech, and abnormal motor behavior. Structural and functional changes occur in the brain due to various chemical alterations in the schizophrenic patient. These changes adversely impact behavioral, emotional and cognitive capabilities of a patient. Schizophrenia patients often experience deterioration or impairment in working memory (WM) ^1^. WM is a short-term memory of a person for perceiving things that relate to immediate consciousness that helps in language processing, decision making and reasoning ^2, 3^. It is an active and readily accessible mental state that maintains information and processes the information selectively ^4^.

The use of functional magnetic resonance imaging (fMRI) has facilitated the diagnosis and treatment of neurological and psychological disorders and enhanced our understanding of the brain. The blood oxygenation level-dependent (BOLD) technique has been widely used in fMRI studies; it relies on the effect of magnetic susceptibility of deoxyhaemoglobin. When a brain region is activated by a task, it demands an increased inflow of oxygenated blood and a net increase in signal intensity is observed. Various paradigms such as visual task and auditory oddball task have been designed to find the pattern of functioning of the brain during different cognitive processes. As the Sternberg item recognition paradigm (SIRP) task is a popular working memory task ^5, 6^, we have used the fMRI data of the subjects performing this task.

As fMRI data involves 3-D scans of the whole brain volume across time, it is inherently high dimensional. Independent component analysis (ICA) ^7^ is a popular method that can be applied on fMRI data to produce the temporally coherent brain networks. ICA is a data-driven approach that generates independent components without making any assumptions about the characteristics of the task and time courses. Group-ICA (GICA) is an extension of ICA that helps to analyze group fMRI studies. In this study, ICA is employed to find such independent networks that have significant differences in the regions between healthy subjects and schizophrenic patients affecting the working memory of a person.

In this study, we aim to identify the brain regions, potentially responsible for the working memory dysfunction, using fMRI data involving SIRP task. Towards this end, we have developed a decision model to differentiate between schizophrenia patients and healthy subjects (controls). We have applied group ICA to find the functionally connected components. We have demarcated the brain regions based on Automated Anatomical Labeling (AAL) atlas. In order to carry out the feature extraction, statistical measures are used to evaluate the significance of different regions. Finally, classification guided feature selection is done using support vector machine (SVM) and 1-NN classifiers.

### Related Work

Several psychological, neurological, and computational studies ^1, 6, 8– 12^ have been conducted to identify the pattern of brain activation for different mental tasks in the schizophrenia patient. Park and Holzman ^1^ found that schizophrenia patients suffer a loss in representational processing, leading to working memory deficit. Impairment of performance in working memory tasks such as the Wisconsin Card-Sorting Test (WCST) is an important evidence of the dysfunction of frontal lobe amongst the schizophrenia patients. Gold *et al*. ^11^ studied the effect of schizophrenia on working memory dysfunction by performing WCST and letter-number (LN) span test on a group of 36 patients with schizophrenia and 30 healthy controls. They found that patients with schizophrenia showed poor performance on the WCST and LN span test, indicating the failure of working memory, typically attributed to frontal lobe dysfunction. Bertilino *et al*. ^8^ performed WCST on a population of 13 patients with schizophrenia and an equal number of healthy subjects to identify the relationship between neuronal pathology of the dorsolateral prefrontal cortex (DLPFC) and activation of working memory network in the cortical region. They found that the rate of N-acetylaspartate level in the DLPFC was firmly linked with the activation of the working memory cortical network during the working memory tasks in schizophrenia patients.

Some researchers experimented with other visual tasks like Sternberg Item Recognition Paradigm (SIRP) to evaluate the impact of schizophrenia on the working memory. Manoach *et al*. ^6^ performed the SIRP task on 12 schizophrenic and 10 healthy subjects. Using SIRP task in fMRI, they compared the activation of DLPFC between the patients and the healthy subjects. A high working memory load condition was compared with non-working memory condition as well as with low working memory load condition. They found that schizophrenia patients performed poorly in comparison to the healthy subjects under different load conditions. They also noted increased DLPFC activation in schizophrenics in comparison to healthy subjects during WM task. In another study, Manoach *et al*. ^13^ examined the participation of brain regions in WM performance by analyzing region-wise brain activations in fMRI data from nine schizophrenic subjects and an equal number of healthy subjects while performing a modified version of the SIRP task, which included a cash reward for correct responses. Again, they compared the high and the low working memory load conditions to each other, keeping the non-working memory condition as a baseline. It was seen that schizophrenic patients showed weak working memory performance along with activation in basal ganglia and thalamus. These regions were found to be activated only in the schizophrenia group. In an fMRI study involving 106 schizophrenic subjects and 111 healthy matched controls, Potkin *et al*. ^12^ examined the BOLD signal change in the DLPFC in a working memory study using SIRP task. They found significantly greater DLPFC activation in patients with schizophrenia. The activation was found to vary with variation in working memory load. The mean BOLD signal was also found to be higher during intermediate memory loads in schizophrenic subjects as compared to the healthy controls. Wible *et al*. ^14^ examined auditory hallucinations while performing the SIRP task in a group of 74 schizophrenic patients, subdivided into non-hallucinating and hallucinating groups. They found that the patients having auditory hallucinations showed decreased functional activity during the probe condition in working memory task mainly in the inferior parietal regions and superior temporal regions in comparison to those not having hallucinations.

ICA treats fMRI data as a linear combination of spatially independent components. These components derived from the fMRI data suggest the functional connectivity between brain regions (also called brain networks). Some of the fMRI studies ^9^ used general linear model (GLM) approach to convert 4D time-series data into a 3D statistical parametric map. Pearson’s correlation coefficient ^15^ and regional homogeneity ^16^ were also applied in fMRI study to extract information from temporal data. Kim *et al*. ^17^ used ICA to trace the temporally coherent networks in fMRI activity using a working memory task. Using the fMRI dataset for 115 patients with chronic schizophrenia and 130 healthy controls performing the SIRP task, they identified six components mainly showing disease-relevant brain networks. These components showed the regions that exhibited significant differences in the functioning of WM networks between schizophrenic patients and healthy controls. Two out of the six networks showed regions covering working memory areas such as bilateral DLPFC, inferior parietal lobules and cerebellum. They observed dysfunction in default mode network (DMN) in schizophrenia which exists across multiple subnetworks in the region. Correa *et al*. ^10^ also explored the role of ICA in the analysis of fMRI data. They compared the performance of different ICA algorithms and performed an analysis of fMRI data having visual-motor task and estimated activations using Infomax, FastICA, eigenvalue decomposition (EVD) and joint approximate diagonalization of eigen matrices (JADE). The authors concluded that the infomax performed quite well on the fMRI data and showed the highest t-values and successfully estimated maximally independent components.

## Methods

### Dataset

The fMRI data used in this study were downloaded from the Functional BIRN Data Repository ( http://fbirnbdr.birncommunity.org:8080/BDR/) ^12^. A detailed description of the data is available at the repository. In brief, all the acquisitions were carried out using 1.5T scanners keeping all other parameters same for all the subjects across the datasets. In this study, we have considered SIRP task fMRI data available at site 0009 and site 0010 of the FBIRN repository. All the three runs of each subject’s scan are used in our experiments. All subjects had regular hearing levels and sufficient eyesight to perform the SIRP task. They were able to perform the cognitive task. Healthy subjects were excluded if they had a current or past history of head injury and major medical illness. All the healthy subjects were free from any antipsychotic exposure and they had no recent history of medication effect. fMRI data from the patients with schizophrenia and schizoaffective disorder meeting the Diagnostic and Statistical Manual of Mental Disorders, 4th edition (DSM-IV) criteria were included in this study. FBIRN had determined the symptom scores by using the Schedule for the Assessment of Positive Symptoms (SAPS) and Negative Symptoms assessment measures ^5^. Table 1 summarizes the database details.

**Table 1.** Demographic details of the dataset.

Subject	No. of subjects	Age group, years *	Sex ratio, male/female
Healthy	34	40.4 ±12.29	24/10
Schizophrenia	34	40.3 ±10.89	17/17

*Data given as mean ± standard deviation.

### Imaging parameters

The functional scans were acquired using T2*-weighted gradient echo planar imaging (EPI) sequences and were parameterized by Orientation: anterior commissure-posterior commissure line; the number of slices: 27; slice thickness: 4 mm; TR: 2 seconds; time to echo: 40 ms; matrix: 64 × 64; field of view: 22 cm; and flip angle: 90° ^12^.

### Task details

In this paper, we have considered the Sternberg item recognition paradigm (SIRP) task ^18, 19^. The SIRP is a block design task that assesses the maintenance and scanning components of WM ^4, 19^. Each phase began with the presentation of a memory set composed of one, three, or five digits, constituting three levels of WM load (low 1L, medium 3L, high 5L). This encode phase was followed by the presentation of 14 probe digits. Participants responded to each probe using a button box to indicate whether the probe digit was in the memory set. Each of the three runs contained two blocks of each of the three load phases, presented in a pseudorandom order with the blocks of each phase alternating with fixation epochs (a baseline resting period). Each run lasted for 6 minutes.

### Data preprocessing

For preprocessing the raw fMRI datasets taken from FBIRN repository, we have used the Statistical Parametric Mapping version 8 (SPM8, Wellcome Trust Centre for Neuroimaging, University College London, UK) ^20^ toolbox in Matlab. The preprocessing steps are as follows. Realignment and reslicing were performed on each of the images using the default parameters. Slice timing correction was applied to correct possible errors introduced by temporal variations during the acquisition of fMRI data. Subsequently, the fMRI scans were spatially normalized into the standard Montreal Neurological Institute (MNI) space using an EPI template. Thus, the volume of each voxel in raw fMRI scans changed from 3.4 × 3.4 × 4 mm ^3^ to 3 × 3 × 3 mm ^3^. This resulted in a brain volume of 53 × 63 × 46 voxels. Finally, spatial smoothing was done using a 9 × 9 × 9 mm ^3^ full width at half-maximum (FWHM) Gaussian kernel on the normalized volumes to get the smoothed volumes.

### Proposed approach

**Figure 1.**
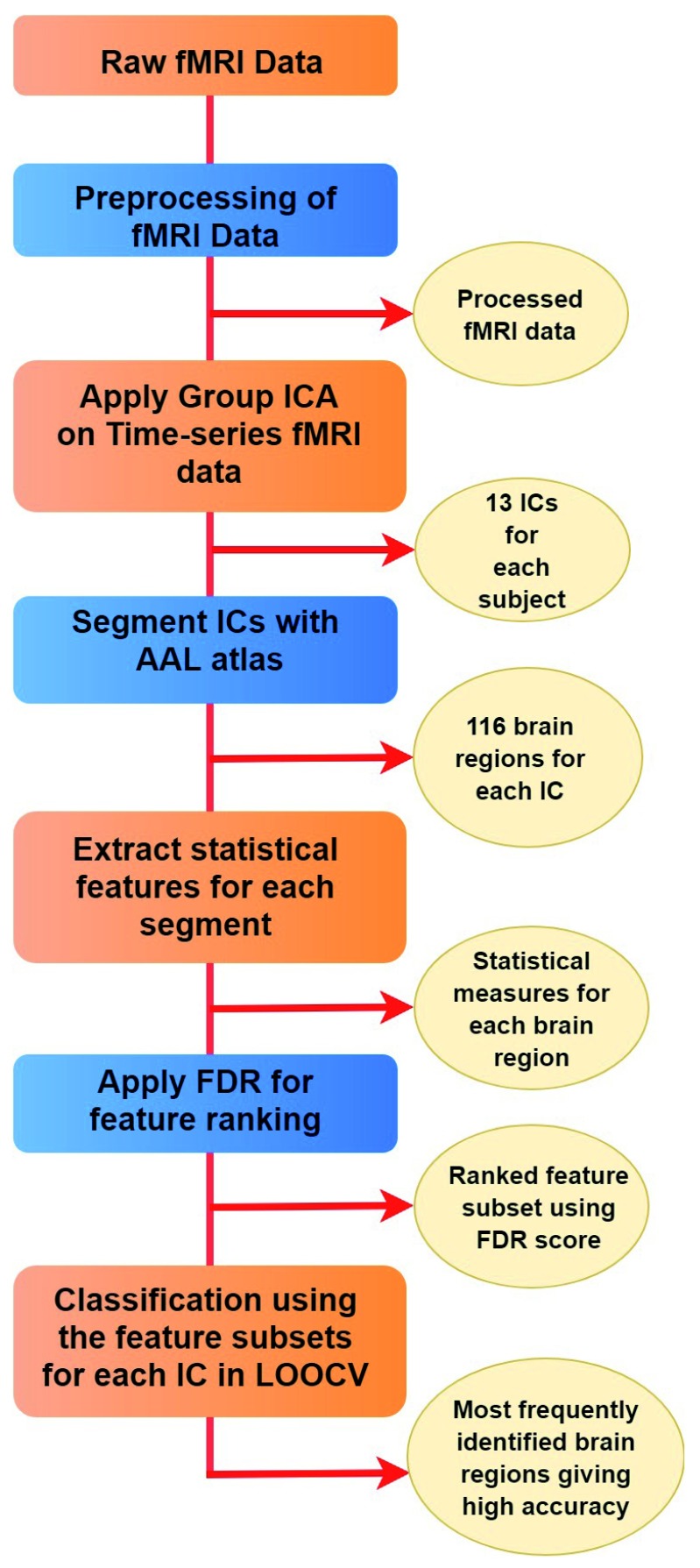
Stepwise representation of the proposed approach.

The proposed approach is divided into the following phases: (i) application of group ICA; (ii) statistical feature extraction; (iii) classification guided feature selection; and (iv) visualization. These phases are described in the following sub-sections. The proposed approach is applied to individual ICs. The stepwise description of the proposed approach is outlined in Algorithm 1. Figure 1 shows the overall workflow of the study.

Algorithm 1. **The proposed approach**

1. Application of group ICA:

(a) Apply GICA on the pre-processed fMRI data, where, the modified MDL criteria is used to identify the number of IC.

2. Feature Extraction:

(a) Segment each IC for each subject in 116 regions using AAL atlas.
(b) Extract five statistical features namely, mean, standard deviation, kurtosis, skewness and entropy from each region for every subject on the basis of voxel values of that particular region. Thus a subject is represented as 580 features (=116 x 5). The dataset is represented as
${\vec{X}}_{68\times 580}=[{\vec{f}}_{1}\;{\vec{f}}_{2}\;{\vec{f}}_{3}\;\cdots \;{\vec{f}}_{580}],$ where ${\vec{f}}_{1}$ is the *i ^th^* feature.

3. Feature Selection:

(a) Carry out feature selection in LOOCV manner. In *i ^th^* fold of LOOCV, all, but *i ^th^* sample is used for training.
(b) Compute FDR score for each feature using equation mentioned in Section 4.2.4.
(c) Rank the features on the basis of FDR score (the feature with highest FDR score is assigned rank 1).
(d) Build decision model (DM) incrementally (forward feature selection) using SVM classifier. Begin by building the DM with the first ranked feature and add the FDR ranked features, one by one, to obtain the high classification accuracy.

4. Visualization:

(a) Identify the set of features having maximum classification accuracy.
(b) Backtrack the features to the MNI brain space to locate the affected brain regions.

### Application of ICA

The BOLD fMRI technique acquires 3-D brain volumes across time. Each voxel in the whole brain volume contains a value that corresponds to the change of signal intensity of the voxel across time. To identify the connected brain networks that are activated while performing a task, we applied group ICA (GICA) using the GIFT toolbox v.4.0b ^21^ in MATLAB. There are three main stages in GICA: data compression (also called data reduction), ICA, and back reconstruction. In the data compression step, principal components analysis (PCA) is used to reduce the size of the data. Group PCA is applied to all subjects. Then, ICA is used to find the independent components (ICs) and the spatial maps. Although several ICA algorithms, such as Infomax, FastICA, Jade, and AMUSE, are available in the GIFT toolbox, we use the most widely used Informax algorithm to find the ICs. The Infomax algorithm ^22^ uses a non-linear function to maximize the information transfer from the input layer to the output layer of a network. The components resulting from ICA represent the brain networks activated during the task. The back-reconstruction step produces the ICs with the most accurate spatial maps and time courses for each subject.

In our experiments, the number of ICs was estimated using the modified minimum description length (MDL) ^23^ criteria, which generated 13 ICs. We have used the average ICs spatial map for each subject corresponding to the three runs. In the proposed approach, we have analyzed 13 ICs independently for each subject.

### Segmentation of ICs

In the first phase, we have segmented each of the 13 ICs for each subject using Automated Anatomical Labeling (AAL) ^24^ atlas. AAL atlas segments the whole brain volume into 116 brain regions. Thereafter, subject-wise features were extracted from each of these 116 regions for each IC.

### Feature extraction

For dimensionality reduction, we have extracted five statistical features for each brain region of each subject, namely, mean, standard deviation (std), skewness, kurtosis, and entropy. If *V _r_* = [ *v*_1_, *v*_2_, *v*_3_, ..., *v*_N_] is the voxel set having *N* voxels for *r ^th^* region, then these statistical measures are defined as follows:

$$Mean\left(\mu_{r}\right)=\frac{\Sigma_{i=1}^{N}v_{i}}{N}$$

$$StandardDeviation\left(\sigma_{r}\right)=\sqrt{\frac{\Sigma_{i=1}^{N}{\left(v_{i}-\mu_{Vr}\right)}^{\text{2}}}{N}}$$

$$Skewness\left(S_{r}\right)=\frac{\frac{1}{N}\Sigma_{i=1}^{N}{\left(v_{i}-\mu_{Vr}\right)}^{3}}{\sigma^{3}}$$

$$Kurtosis\left(K_{r}\right)=\frac{\frac{1}{N}\Sigma_{i=1}^{N}{\left(v_{i}-\mu_{Vr}\right)}^{4}}{\sigma^{4}}$$

$$Entropy\left(E_{r}\right)=-\Sigma_{i}\left(P_{i}\right).\log_{2}\left(P_{i}\right)\;where\;P_{i}=Histogram\left(V_{r}\right)$$

Thus, for each subject, we extracted 580 (= 116 × 5) features. To identify the features relevant for identifying affected brain regions in schizophrenia, we carried out feature selection.

### Classification guided feature selection

Feature selection is a process of selecting a relevant subset of the feature set. In this paper, we have incorporated the classification guided sequential forward feature selection method in a leave-one-out cross-validation (LOOCV) manner. In the sequential forward selection, one adds the best features in every iteration, until the best classification accuracy is achieved. We have used Fisher’s discriminant ratio (FDR) score for ranking each feature. FDR score was computed using the formula,

$$FDR\;Score\;(x)=\frac{|mean_{h}-mean_{s}{|}^{2}}{var_{h}+var_{s}}$$

where

*x* = vector containing the feature *x* values corresponding to all subjects,
*mean _h_* = mean of feature *x* corresponding to healthy patients;
*mean _s_* = mean of feature *x* corresponding to schizophrenic patients;
*var _h_* = variance of feature *x* corresponding to healthy patients;
*var _s_* = variance of feature *x* corresponding to schizophrenic patients.

After scoring all the features, the scores were arranged in descending order. The high value of FDR indicates that the within-class scatter is low, while between-class scatter/variance is high. Forward feature selection approach is employed to identify the feature set generating high classification accuracy. 

### Classification

Several works ^9, 25– 27^ have attempted to identify the affected brain regions using a decision model to classify schizophrenia patients and healthy controls. In this paper, classification task is performed on the combined data involving the healthy and schizophrenic subjects using linear SVM and k - nearest neighbors (k-NN) classifiers. Classification is done in LOOCV manner i.e., training is done on all the subjects excluding one subject, which is used for testing. The classification model is built incrementally. Finally, the feature subset yielding high classification accuracy for a given test sample is chosen.

Each sample is an input vector *X _i_* ( *i* = 1, 2, 3, ..., n) having features selected from the statistical measure of a particular region (set of voxels) and is associated with one of the two classes *Y _i_* = +1 or *Y _i_* = -1 (binary class). The class labels +1 and -1 refers to the positive class (schizophrenia) and the negative class respectively. For classification using SVM, the libsvm version 3.23 ^28^ package in Matlab-2014b is used that uses C-SVC. Besides setting all the training parameters as default, we experiment with the different values of cost parameter ( *C*), varying *C* in the range of 0.01 to 1000 in powers of 10. For *k*-NN classifier, we take the value of *k* as 1 and use Euclidean distance as the distance metric.

### Visualization

The set of selected features, obtained after all the iterations of LOOCV approach for each IC, were backtracked to brain space to identify the affected brain regions. In order to find the most relevant regions that may contribute to the dysfunction of the working memory in the schizophrenia patients, the brain regions identified by the proposed approach, marked by different independent components, were coalesced. The frequently occurring regions were plotted on a mask using WFU PickAtlas. The mask was then overlaid onto a standard T1-weighted MRI using MANGO version 4.0.1 toolbox ^29^. These identified brain regions were overlaid onto a standard *T*-1 weighted image and visualized MANGO toolbox.

## Results

**Figure 2.**
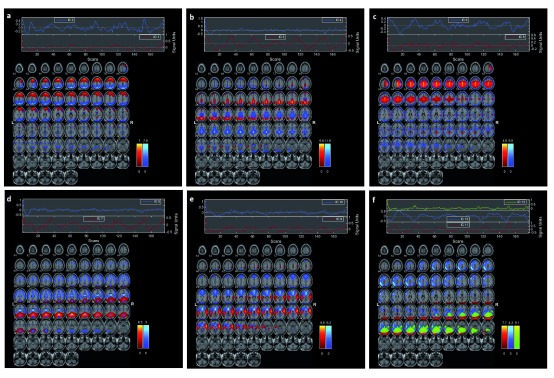
Composite images showing the 13 independent components across all the subjects. The connected brain networks identified by ( **A**) independent component (IC) 1–IC 2, ( **B**) IC 3–IC 4, ( **C**) IC 5–IC 6, ( **D**) IC 7–IC 8, ( **E**) IC 9–IC 10, and ( **F**) IC 11–IC 13, are shown.

**Figure 3.**
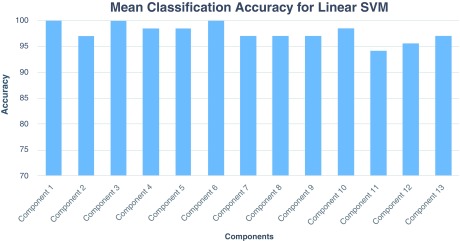
Mean classification accuracy with linear SVM classifier for each independent component across 10 different runs.

**Figure 4.**
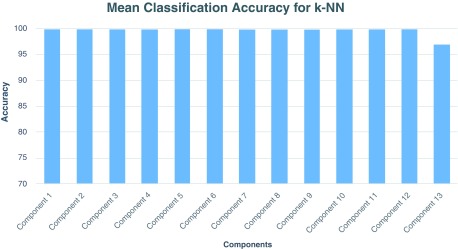
Mean classification accuracy with k-NN classifier (k=1) for each independent component across 10 different runs.

**Figure 5.**
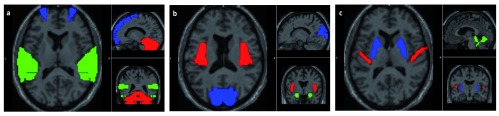
The most distinct regions identified in brain responsible for dysfunction in schizophrenia are shown. ( **A**) shows the cerebellum (red), inferior and superior temporal gyrus (green), and superior frontal gyrus (blue); ( **B**) shows the insula (red), amygdala (green), and cuneus (blue); ( **C**) shows the Heschl gyrus (red), vermis (green) and putamen (blue).

The first phase of the proposed method resulted in 13 spatial ICs (see Figure 2). Figure 2a–f shows the composite view of multiple independent components showing functionally connected brain regions involved during the task. The figure highlights task-related components with functional differences across healthy and schizophrenia subjects. We have used the forward feature selection method in the second phase. The average classification accuracy using LOOCV scheme for SVM and *k*-NN classifiers for each IC is shown in Figure 3 and Figure 4, respectively. Overall, the linear SVM classifier ( *C*=1.09) yielded classification accuracy in the range 94% to 100%. Similarly, the use of *k*-NN classifier resulted in classification accuracy in the range of 96–100% (see Figure 4 for linear SVM and Figure 5 for 1-NN classifier). Finally, the affected brain regions identified from the visualization phase are mentioned in Table 2 for each spatial IC. Table 2 shows the regions marked by increased activation in case of schizophrenia patients when compared to the healthy controls. Figure 5 (a–c)) shows the identified regions such as the cerebellum, temporal and frontal gyrus, insula, amygdala, cuneus, putamen, Heschl gyrus, and vermis.

**Table 2.** Brain regions identified by the proposed approach for each independent component (IC).

IC 1	IC 2	IC 3	IC 4	IC 5	IC 6	IC 7	IC 8	IC 9	IC 10	IC 11	IC 12	IC 13
Amygdala_L	Amygdala_L	Amygdala_L	Amygdala_R	Amygdala_L	Amygdala_L’	Amygdala_L	Amygdala_L	Amygdala_R	Amygdala_R	Amygdala_R	Amygdala_L	Amygdala_L’
Amygdala_R	Angular_L	Angular_L	Angular_R	Angular_L	Angular_L	Angular_L	Angular_R	Angular_R	Angular_R	Calcarine_L	Angular_R	Angular_L
Angular_L	Calcarine_L	Calcarine_R	Calcarine_R	Calcarine_L	Caudate_R	Calcarine_R	Calcarine_R	Calcarine_R	Caudate_L	Caudate_R	Calcarine_R	Cerebelum_10_L
Cerebelum_ 10_L	Caudate_L	Cerebelum_10_R	Cerebelum_10_L	Caudate_R	Cerebelum_10_R	Caudate_R	Caudate_L	Cerebelum_9_R	Cerebelum_10_R	Cerebelum_ 10_L	Caudate_R	Cerebelum_Crus1_L
Cerebelum_ 10_R	Cerebelum_10_L	Cerebelum_Crus1_L	Cerebelum_Crus1_L	Cerebelum_ 10_L	Cerebelum_ Crus1_R	Cerebelum_10_L	Cerebelum_10_L	Cerebelum_ Crus2_R	Cerebelum_Crus1_L	Cerebelum_6_R	Cerebelum_10_L	Frontal_Inf_ Oper_L
Cerebelum_6	Cerebelum_Crus1_L	Cingulum_Ant_L	Cingulum_Ant_L	Cerebelum_ 3_L	Cingulum_Ant_R	Cerebelum_Crus1_R	Cerebelum_ Crus1_R	Cingulum_Post_R	Cuneus_L	Cerebelum_ Crus1_L	Cerebelum_6_R	Frontal_Med_Orb_L
Cerebelum_ 6_R	Cingulum_Ant_L	Cingulum_Mid_L	Cingulum_Post_R	Cingulum_Ant_L	Cuneus_R	Cuneus_L	Cingulum_ Ant_L	Cuneus_R	Frontal_Inf_ Oper_L	Cingulum_Ant_R	Cerebelum_ Crus1_L	Frontal_Mid_R
Cerebelum_ 9_L	Cingulum_Mid_L	Cingulum_Post_L	Cuneus_L	Cuneus_R	Frontal_Inf_Orb_L	Frontal_Med_Orb_L	Cingulum_Post_L	Frontal_Inf_Tri_L	Frontal_Inf_Tri_R	Cuneus_R	Cingulum_Ant_R	Frontal_Sup_ Medial_R
Cerebelum_ Crus1	Cingulum_Post_L	Cuneus_R	Frontal_Inf_Oper_L	Frontal_Inf_ Oper_L	Frontal_Mid_L	Frontal_Mid_Orb_L	Cingulum_Post_R	Frontal_Mid_ Orb_L	Frontal_Mid_Orb_R	Frontal_Inf_Oper_L	Frontal_Inf_Orb_R	Hippocampus_L
Cuneus_R	Cuneus_L	Frontal_Inf_Oper_L	Frontal_Med_Orb_R	Frontal_ Med_Orb_L	Frontal_Sup_ Medial_L	Frontal_Sup_L	Cuneus_R	Frontal_Sup_R	Fusiform_L	Frontal_Med_Orb_R	Frontal_Med_Orb_L	Insula_L
Frontal_ Med_Orb_L	Frontal_Inf_Oper_L	Frontal_Med_Orb_L	Frontal_Sup_R	Frontal_Mid_ Orb_L	Heschl_R	Fusiform_R	Frontal_Inf_ Oper_L	Fusiform_R	Heschl_L	Frontal_Sup_L	Frontal_Mid_Orb_L	Lingual_L
Frontal_Sup_ Medial_R	Frontal_Inf_Orb_L	Frontal_Mid_L	Fusiform_L	Fusiform_L	Hippocampus_R	Heschl_R	Frontal_Inf_Tri_R	Heschl_L	Heschl_R	Fusiform_R	Frontal_Sup_L	Occipital_Inf_L
Frontal_ Sup_R	Frontal_Med_Orb_L	Frontal_Sup_L	Heschl_R	Heschl_L	Occipital_Mid_R	Lingual_R	Frontal_Med_ Orb_R	Hippocampus_R	Hippocampus_R	Heschl_R	Fusiform_R	Occipital_Sup_R
Fusiform_R	Frontal_Mid_L	Fusiform_L	Hippocampus_R	Insula_R	Olfactory_L	Occipital_Mid_R	Frontal_Sup_L	Insula_R	Lingual_R	Hippocampus_R	Heschl_R	Olfactory_L
Heschl_L	Frontal_Sup_L	Heschl_R	Insula_R	Olfactory_L	Parietal_Sup_R	ParaHippocampal_R	Fusiform_R	Occipital_Inf_R	Occipital_Inf_L	Insula_L	Hippocampus_R	Pallidum_R
Insula_L	Fusiform_L	Hippocampus_R	Occipital_Inf_L	Pallidum_L	Precuneus_R	Parietal_Inf_L	Heschl_L	Occipital_Sup_R	Occipital_Sup_R	Occipital_Inf_R	Insula_L	Paracentral_ Lobule_R
Occipital_ Inf_R	Heschl_L	Insula_L	Pallidum_R	Paracentral_ Lobule_R	Rectus_R	Precuneus_R	Hippocampus_R	Pallidum_R	Paracentral_ Lobule_R	Pallidum_L	Occipital_Inf_L	Parietal_Sup_R
Occipital_ Sup_R	Hippocampus_R	Lingual_L	Paracentral_Lobule_R	Postcentral_L	Temporal_Inf_L	Putamen_R	Insula_L	Paracentral_ Lobule_R	ParaHippocampal_R	Paracentral_ Lobule_L	Occipital_Sup_R	Precuneus_L
Precuneus_L	Insula_L	Occipital_Inf_R	ParaHippocampal_L	Putamen_R	Temporal_Mid_R	Supp_Motor_Area_R	Insula_R	Parietal_Sup_L	Parietal_Inf_L	Parietal_Inf_R	Olfactory_L	Putamen_R
Temporal_ Inf_R	Lingual_L	Occipital_Mid_L	Parietal_Sup_L	Rectus_L	Thalamus_L	SupraMarginal_R	Occipital_Inf_L	Postcentral_L	Postcentral_R	Precuneus_R	Paracentral _Lobule_L	Rectus_L
Temporal_ Pole_Mid_R	Occipital_Inf_L	Occipital_Sup_L	Putamen_L	Rectus_R	Vermis_1_2	Temporal_ Pole_Mid_L	Occipital_Sup_R	Putamen_R	Precuneus_R	Putamen_R	ParaHippocampal_L	Supp_Motor _Area_L
Vermis_1_2	Occipital_Mid_L	Olfactory_R	Supp_Motor_Area_R	Temporal_ Inf_L		Thalamus_L	Pallidum_R	Rolandic_Oper_L	Putamen_L	Rectus_R	Parietal_Sup_R	SupraMarginal_R
Vermis_6	Occipital_Sup_R	Pallidum_R	Temporal_Inf_L	Thalamus_R		Vermis_3	Paracentral_ Lobule_L	Supp_Motor_ Area_R	Rectus_L	SupraMarginal_L	Postcentral_L	Temporal_Inf_L
	Olfactory_L	Paracentral_ Lobule_R	Thalamus_L	Vermis_1_2			Parietal_Inf_L	SupraMarginal_R	Rolandic_Oper_R	Temporal_Mid_R	Precuneus_L	Temporal_Mid_L
	Pallidum_L	ParaHippocampal_R	Vermis_3				Precentral_L	Temporal_Mid_L	SupraMarginal_L	Temporal_Sup_R	Putamen_R	Temporal_Sup_R
	Paracentral_Lobule_L	Parietal_Inf_R					Precentral_R	Temporal_Sup_R	Temporal_Inf_L	Thalamus_L	Rectus_L	Thalamus_L
	ParaHippocampal_L	Parietal_Sup_R					Supp_Motor_Area_R	Thalamus_R	Temporal_Pole_Mid_L	Vermis_1_2	Supp_Motor_Area_R	Vermis_1_2
	Parietal_Inf_L	Precentral_L					SupraMarginal_L	Vermis_1_2	Temporal_Sup_L	Vermis_6	SupraMarginal_L	Vermis_3
	Parietal_Sup_L	Precuneus_L					Temporal_Inf_R	Vermis_10	Vermis_1_2		Temporal_Mid_R	Vermis_6
	Postcentral_L	Putamen_L					Temporal_Sup_R	Vermis_6	Vermis_6		Temporal_Sup_L	
	Precentral_L	Rolandic_Oper_L					Thalamus_L				Thalamus_L	
	Precuneus_R	Supp_Motor_Area_R					Vermis_3				Vermis_1_2	
	Putamen_L	SupraMarginal_R					Vermis_6				Vermis_6	
	Rolandic_Oper_L	Temporal_Inf_L										
	Supp_Motor_Area_L	Temporal_Mid_R										
	SupraMarginal_L	Thalamus_R										
	Temporal_Inf_L	Vermis_1_2										
	Temporal_Mid_L											
	Thalamus_L											
	Vermis_1_2											

## Discussion

This study aimed to identify affected brain regions in the working memory of schizophrenia patients. To achieve this, we proposed a model wherein we utilized the GICA to obtain spatial ICs, extracted statistical features from 116 brain regions, selected features using a classifier-guided forward feature selection approach, and visualization of affected brain regions. Using the proposed approach, we marked the differences in the functional activation of the following brain regions in most of the ICs (the cerebellum, inferior temporal gyrus, superior temporal gyrus, superior frontal gyrus, Heschl gyrus, insula, amygdala, vermis, thalamus, calcarine, occipital lobe and hypocampus) in schizophrenia patients in comparison to healthy controls. It may be noted that the brain regions identified in this study are largely in conformity with the previous studies ^30– 34^. Further, the connected brain regions discovered by the different spatial ICs, largely confirm to each other.

Our results show the functional changes in the cerebellum region. Previous studies ^32, 33^ also suggest some changes in cortical cerebellar regions and its functional connectivity in working memory performance in schizophrenia patients. We found functional changes in the inferior temporal gyrus, superior temporal gyrus and superior frontal gyrus. While earlier studies ^32, 35, 36^ suggest changes in activation and abnormal functional connectivity in temporal and frontal gyri in schizophrenia patients compared to healthy subjects, our results show changes in the Heschl gyrus region. In support of our finding, we may note that in a study by Hirayasu *et al*. ^30^, they found structural volume reduction in Heschl gyri in schizophrenia patients. Grey matter atrophy in Heschl gyri was also found in a study by Kasai *et al*. ^31^. In this regard, we may say that the study of relationships between the functional activations in the relating to working memory dysfunction and structural brain changes in Heschl gyrus could be an important direction of research. We find significant changes in the insula region of the schizophrenic brain similar to the result of other previous studies ^37, 38^. Our results also show functional changes in the amygdala region. While performing working memory tasks, evidence of the dysfunction or abnormalities in the amygdala in schizophrenia patients were found in several previous studies ^39– 42^. We also found some changes in the functional activation in the vermian area, specifically in cerebellar vermis. Although, some literature ^43, 44^ reports structural changes in the vermis region, our study may be a cue for further research based on ROI study to trace the changes in this region associated with schizophrenia and working memory task.

In addition, we obtained a high classification accuracy (>95%) to distinguish healthy subjects and schizophrenic. The high classification accuracy obtained by applying the proposed approach proves its efficacy in comparison to the other fMRI studies ^17, 45, 46^. Overall, the proposed approach found to be effective and efficient in the identification of affected brain regions responsible for working memory dysfunction in schizophrenia.

## Conclusion

In this fMRI study, based on working memory task, a feature selection scheme has been proposed to identify the brain regions affected amongst the schizophrenia patients. This study helps in the identification of brain regions responsible for impairment of working memory in schizophrenia patients. While many connected brain regions identified in our study confirm the findings of the previous studies, the results reveal some new regions in the brain which have not been reported till date in the working memory literature for schizophrenia. These regions may play role in dysfunction of working memory in the patients and could be the subject of further studies.

## Data availability

The SIRP task fMRI data from the FBIRN phase II repository can be downloaded from http://schizconnect.org/queries/new, querying 1.5T fMRI data for healthy and schizophrenia subjects available at site 0009 and 0010. The list of subjects chosen for this study is mentioned in the ‘dataset_SubjectID_list.txt’ file available with the codes. Users are required to sign-up to SchizConnect to download data; conditions of use are as written in the data use agreement of the FBIRN project.

To download the data used in this study, the user has to select the project as ‘Study: fBIRNPhaseII__0010’, add ‘AND’, and select MRI as ‘Field Strength: 1.5’.

## Software availability

**The complete source code is archived at:** https://doi.org/10.5281/zenodo.2528773 ^47^.

**License:** Creative Commons Zero v1.0 Universal.
