# Supplementary material for: Identification of brain regions associated with working memory deficit in schizophrenia
Source: F1000Res. 2019 Jan 30;8:124. [Version 1] doi: 10.12688/f1000research.17731.1 (PMC6480944; doi:10.12688/f1000research.17731.1)
Supplement: Peer review history

# Peer review: Reviewer response for version 1

**DOI:** 10.5256/f1000research.19389.r45564

**Published:** 2019-04-18

**Authors:** Lovekesh Vig

**Affiliations:** 1 TCS Research, New Delhi, Delhi, India

The paper presents techniques to identify regions in working memory affected by schizophrenia from fMRI data. The procedure followed is methodical but the claims to identify previously unidentified brain regions need clinical verification. The classification is yielding impressive results but the dataset is too small to be conclusive, however I understand how difficult it is to obtain fMRI data for schizophrenic patients. Overall, I am satisfied with the technical contribution in this article.

I have read this submission. I believe that I have an appropriate level of expertise to confirm that it is of an acceptable scientific standard.

# Peer review: Reviewer response for version 1

**DOI:** 10.5256/f1000research.19389.r43805

**Published:** 2019-02-21

**Authors:** Nipa Roy

**Affiliations:** 1 School of Physics, Centre of Excellence for Integrative Brain Function, University of Sydney, Sydney, NSW, Australia

The authors have revealed some important brain regions active for schizophrenia patients in addition to some other reported regions in previous studies. This will give some clinical advantages in this certain brain disease. However, this whole study is based on the dysfunction of working memory, and a portion of fMRI data set. The authors did not emphasized enough on the selection criteria of elimination of certain region of the same data set. Furthermore, are the authors thinking not to generalize their finding of “some functional changes in the brain regions, such as Heschl gyrus and the vermian area” at this point (in this work) due to the sample size they are taking into account?

I have read this submission. I believe that I have an appropriate level of expertise to confirm that it is of an acceptable scientific standard.
